# Determinants of dog owner-charged rabies vaccination in Kinshasa, Democratic Republic of Congo

**DOI:** 10.1371/journal.pone.0186677

**Published:** 2017-10-23

**Authors:** Eric Kawaya Kazadi, Georges Mbuyi Tshilenge, Victor Mbao, Zakariaou Njoumemi, Justin Masumu

**Affiliations:** 1 Veterinary Faculty, University of Kinshasa, Kinshasa, Democratic Republic of Congo; 2 Veterinary Laboratory of Kinshasa, Kinshasa, Democratic Republic of Congo; 3 International Development Research Centre, Nairobi, Kenya; 4 Faculty of Medecine and Biomedical Sciences, University of Yaounde 1, Yaounde, Cameroon; 5 Veterinary Faculty, National Pedagogical University, Kinshasa, Democratic Republic of Congo; 6 National Institute for Biomedical Research, Kinshasa, Democratic Republic of Congo; University of Minnesota College of Veterinary Medicine, UNITED STATES

## Abstract

Rabies is a preventable fatal disease that causes about 61,000 human deaths annually around the world, mostly in developing countries. In Africa, several studies have shown that vaccination of pets is effective in controlling the disease. An annual vaccination coverage of 70% is recommended by the World Health Organization as a control threshold. The effective control of rabies requires vaccination coverage of owned dogs. Identification of the factors determining dog owners’ choice to vaccinate is necessary for evidence-based policy-making. However, for the Democratic Republic of Congo (DRC), the limited data on rabies vaccination coverage makes it difficult for its control and formulation of appropriate policies. A cross-sectional study was conducted in Kinshasa (Lemba commune) with dog-owning households and owned dogs as study populations. The association between dog vaccination and independent factors (household socio-demographics characteristics, dog characteristics, knowledge of rabies and location of veterinary offices/clinics) was performed with Epi-info 7. The Odds Ratio (OR) and p-value < 0.05 were used to determine levels of significance. A total of 166 households owning dogs and 218 owned dogs were investigated. 47% of the dogs had been vaccinated within one year preceding the survey which is higher than the critical coverage (25 to 40%) necessary to interrupt rabies transmission but below the 70% threshold recommended by WHO for control. The determinants of vaccination included socio-economic level of the household (OR = 2.9, p<0.05), formal education level of the dog owner (OR = 4, p<0.05), type of residence (OR = 4.6, p<0.05), knowledge of rabies disease (OR = 8.0, p<0.05), knowledge of location of veterinary offices/clinics (OR = 3.4, p<0.05), dog gender (OR = 1.6, p<0.05) and dog breed (OR = 2.1, p<0.05). This study shows that the vaccination coverage in this area can easily reach the WHO threshold if supplemented by mass vaccination campaigns.

## Introduction

Rabies is an acute meningoencephalitis caused by a lyssavirus infection. The *lyssavirus* genus belongs to the Mononegavirales order and the Rhabdoviridae family [1]Rabies is mainly transmitted through saliva of infected animals. A wide range of mammals are susceptible and once infected, can transmit rabies through biting other animals. The order of *carnivora* such as domestic dogs (*Canis lupus*), raccoons (*Procyon lotor*), skunks (*Spilogale putorius*), foxes (*Vulpes vulpes*), jackals (*Canis aureus*) and the order of Chiroptera (bats) are considered as reservoirs [2]. The first case of human death due to dog transmitted rabies was documented in 2300 B.C. [3] In Africa, the first rabies oubreak was documented in Algeria in 1858 [4]. In the Democratic Republic of Congo (DRC), the first case of dog rabies was documented in 1923[5]. Rabies is responsible for an estimated 61,000 human deaths per year in the world, predominantly in Asia and Africa [1] Dog to human transmission is responsible for 98% of rabies cases in Asia and Africa [6]

In Kinshasa, the capital of DRC, 11,098 human exposures, 27 confirmed canine cases and 154 humans deaths were recorded between 2003 and 2016 [7,8]. Human and canine cases of rabies are usually under-reported. The disease is preventable and according to WHO [9], the main methods used to control dog rabies include vaccination, culling and sterilization of dogs. Several studies have shown that mass vaccination of domestic dogs is an effective means to control rabies in Africa [10,11]. The WHO vaccination threshold is 70% [1] while the critical coverage required to interrupt rabies transmission ranges between 25–40% [12,13]. Jibat [14] reported that in eleven African countries, rabies vaccination coverage is 18% in the case of owner-charged dog rabies vaccination. In the DRC, rabies vaccination decision making depends entirely of dog owners. The aim of this study was to estimate rabies vaccination coverage of owned dogs and identify the determinants of vaccination in one of 24 communes of Kinshasa.

## Material and methods

### Study area

**Fig 1:**
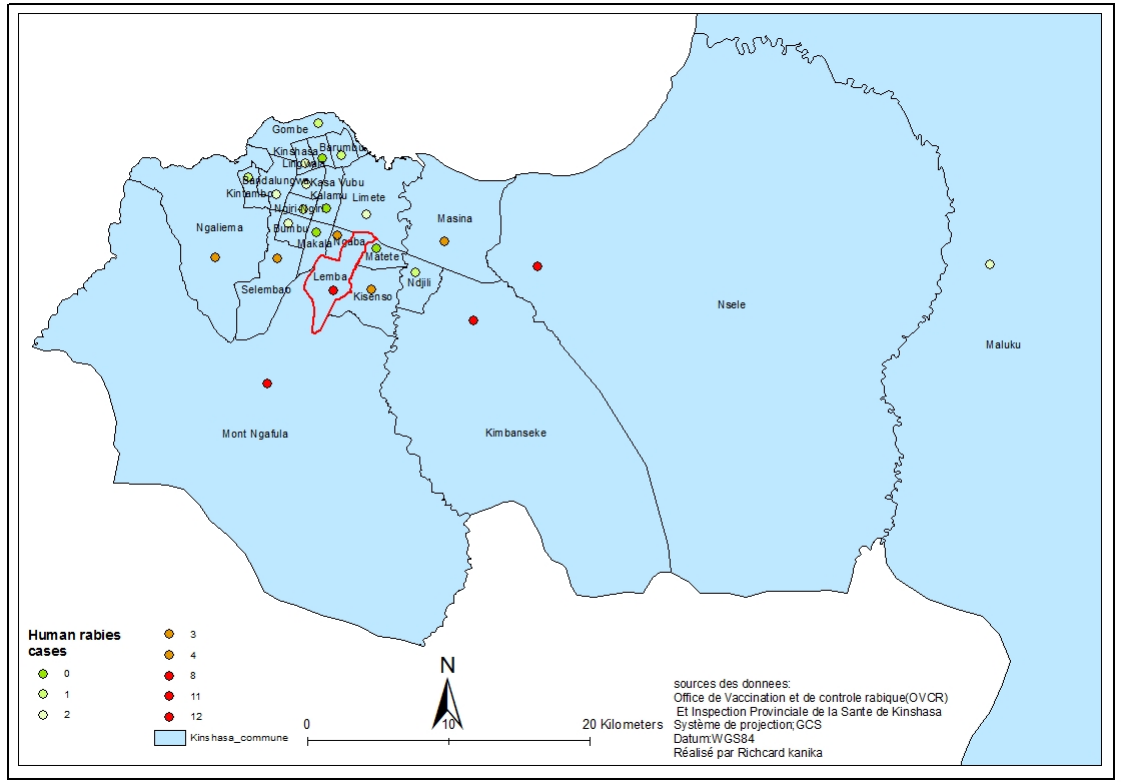
The spatial distribution of human clinical cases of rabies in Kinshasa from 2003 to 2016.

The study was carried out in Lemba, one of the 24 municipalities (communes) of Kinshasa. This municipality is subdivided into 15 areas (quartiers). From 2003 to 2016, no free dog rabies vaccination campaigns were organised in this commune, although some human clinical cases were recorded (Fig 1).

### Cross-sectional study design and data collection

A cross-sectional study was conducted during three months from December 2014 to February 2015 in order to collect data on owned dogs and dog owning households relating to the determinants of dog anti-rabies vaccination following an “owner-charged” scheme in Lemba municipality. In total, 15 areas were investigated with at least 10 dog-owning households selected in each of them. These households were selected through combinations of random and convenience sampling methods as follow: First, a list of the main streets of each of the 15 neighborhoods was established and a number was assigned to each street listed. Two streets were then randomly selected for each of the neighborhoods making a total of 30 selected streets. For each selected street, at least 5 dog owning households were investigated by convenience. The investigation began at the first residence on the left or right side of each street. In these households, any available family member above 18 years of age was interviewed using a semi-structured questionnaire. Information collected included socio-demographics characteristics of household, type of residence, dog characteristics (total/partial confinement or free movement, dog food, dog origin), dog rabies vaccination status, reasons for non-vaccination, knowledge of rabies (transmission and disease outcome, species affected and prevention or control), knowledge of location of veterinary office/clinics. To grade the economic level of the household, we used the following items based on previous studies [15]: durable goods (means of transportation) and housing characteristics (availability of electricity, availability of piped drinking water and number of bedrooms). 0 or 1 score was attributed to each item: 0 in case of usage of public vehicle for transportation, unavailability of electricity, unavailability of piped drinking water, 2 or fewer bedrooms and 1 in case of usage of private vehicle for transportation, availability of electricity, availability of piped drinking water, 3 or more bedrooms. The households scoring a maximum of 2 were classified in the low economic level and those scoring 3 or 4 were classified in the middle economic level. To assess the rabies knowledge of participants, a maximum score of 15was set. Any respondent scoring above 8 was classified as having good knowledge of rabies disease.

### Data analysis

Data were analyzed using Epi-Info version 7 and Ms Excel 2007 to generate frequencies, proportions and graphs. Bivariate analysis of the data was performed using the dog vaccination status (vaccinated and unvaccinated) as the dependent variable and the determinants of vaccination (socio-demographics characteristics of household, dog characteristics and restraint method, dog feeding method, knowledge of rabies, knowledge of location of veterinary office/clinics) as independent variables. Odds Ratio (OR) and p value < 0.05 were used to determine levels of significance. Factors with p-value of ≤ 0.05 were considered as significant [16]. The determination of "dog rabies vaccination" status was based on households and not individual dogs. For households with more than one dog, the vaccination status was "Yes" if at least one of the dogs was vaccinated. The proportion of dog rabies vaccination was based on the number of households with vaccinated dogs.

### Ethical consideration

Approval of the study was obtained from the « Direction de Lutte contre la Maladie (DLM) » a department of the Public Health Ministry of DRC and the ethical clearance from the ethics committee of the Public Health faculty of the University of Kinshasa. Before obtaining the oral consent of participants in the survey, the investigator explained clearly to the participant the objective of the study. They were informed of the choice to participate or not in the survey. The formula “are you willing to participate to this survey?” was used (see first paragraph of the survey questionnaire). Data was coded, backed up in an external disk and protected by the main investigator.

## Results and discussion

A total of 166 dog-owning households and 218 owned dogs were investigated in this study. In total, 60.5% of these dogs had a history of rabies vaccination and 49.5% of them had been vaccinated once within the year leading up to the study while 11% were vaccinated more than once in the same period. In DRC, most dog owners vaccinate their animals with RABISIN^®^ (Merial, France), a monovalent inactivated rabies vaccine widely commercialized in the country. Following the manufacturer's recommendations, effective use of this vaccine requires an annual booster, meaning that 11% of the dogs recorded in this study as having a history of vaccination (but without the annual boost) were not protected. In contrast, 11% had more than one vaccination within the same period showing that they were unnecessarily vaccinated twice. Reasons for this early booster need to be investigated.

The vaccination coverage observed in the current study is below the average dog rabies vaccination coverage (68%) for eleven African countries following a “free of charge” vaccination scheme [14] and below the threshold coverage rate (70%) recommended by WHO [1]. Dog vaccination in DRC is mostly carried out on an "owner-charged" scheme. "Free of charge" vaccination schemes are used from time to time during outbreak periods. Compared to the 18% coverage following an “owner-charged” scheme of dogs rabies vaccination campaigns in eleven African countries[14]), the 49.5% observed in Lemba commune following the same scheme is rather encouraging because this coverage is higher than the critical vaccination coverage (25 to 40%) necessary to interrupt rabies transmission [12]. Additionally, such a high vaccination coverage resulting from the "owner-charged" scheme can easily reach the 70% threshold recommended by WHO if the government could subsidize the price of the vaccination. In Chad, this WHO threshold could not be achieved during a parenteral mass dog vaccination campaign because only 24% of dog owners accepted to pay their 21% of the vaccination costs [17]

**Fig 2:**
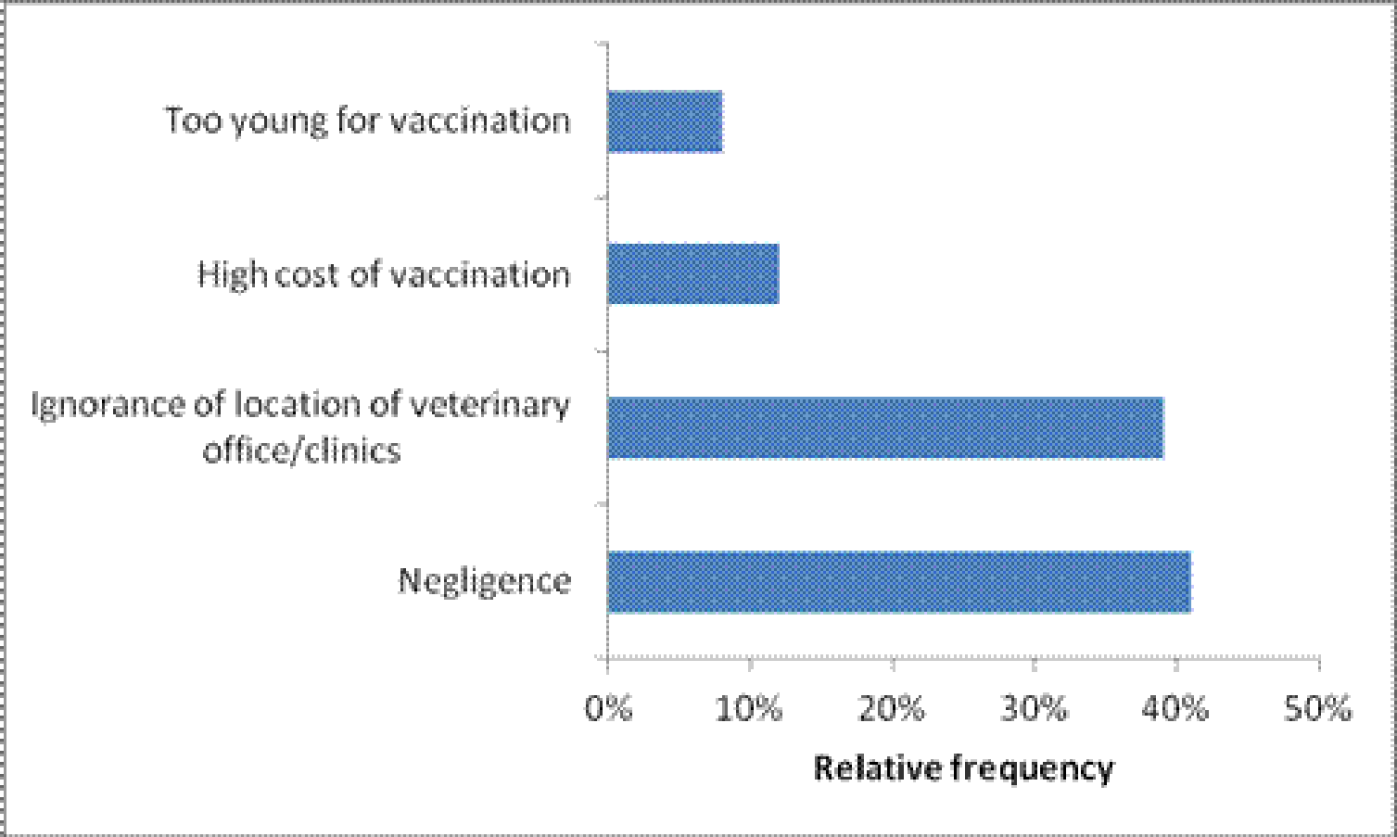
Reasons of non-vaccination of dogs by owners (n = 64) in Lemba commune, Kinshasa/DRC.

Many factors can explain the dog rabies vaccination trends found in the "owner-charged" scheme. These factors can be categorised in three groups: 1) socio-demographic characteristics of the household, 2) knowledge of rabies disease and location of veterinary office/clinics and 3) dog characteristics. In the current study, the socio-demographic factors of the household including the socio-economic level of the household (OR = 2.9, p<0.05), the formal education level of the dog owner (OR = 4, p<0.05) and the type of residence (OR = 4.6, p<0.05) “Table 1” were significantly associated with dog vaccination. According to the study results, the prevalence of vaccinated dogs in middle economic class households was twice higher than in the low economic households. This result could partially be explained by the cost of rabies vaccination that is fixed by the Congolese Veterinary Medical Association to a minimum of USD 20. As shown in “Fig 2”, the cost of rabies vaccination accounted partially for the non-vaccination of dogs in this commune as 12% of the dog-owners considered this amount to be too high. Although some of them presented negligence or lack of knowledge of veterinary clinics as reasons for non-vaccination, it is possible that they were not willing to vaccinate their animals or take them to veterinary clinics because of the high vaccination price. In Chad, the average cost of rabies vaccination campaigns was USD 19.40 per vaccinated dog and owners were charged only 21% (4.13USD) as partial cost recovery. In spite of this, the coverage rate was very low (26%) [6]. These results demonstrate that the cost of rabies vaccination can constrain accessibility of rabies vaccination. This constraint can be overcome if the cost of vaccination is determined by dog-owners’ willingness to pay. In the Philippines, dog-owners were only willing to pay USD 1.67 for dog rabies vaccination [18]. To better assess the variations in the socio-economic determinants of vaccination in the populations living in the rabies endemic areas, such surveys needs to be conducted in other locations in the DRC. This would assist the Veterinary Association of DRC in better estimating the price of rabies vaccine that is affordable in the respective locations. This study showed that dog owners with formal education were more likely to vaccinate their dogs than those without a formal education. This is probably due to access to information on the importance of rabies vaccination.

**Table 1:** Dog-owning households’ characteristics associated to dog rabies vaccination.

	Dog rabies vaccination				
Factors	Yes	No	Proportion (%)	OR	P value
**Socio-economic level**					
Middle	96	54	54	2.9	0.02*
Low	6	10	37.5		
**Formal education level**					
Educated	99	57	63.5	4	0.02
Uneducated	3	7	30		
**Dog owner**					
Household head	70	46	60.3	0.9	0.33
Other person	32	18	64		
**Household dog number**					
More than one dog	22	13	62.9	1.7	0.08
One dog	80	51	61		
**Types of residence**					
Residence with fence	94	46	67.1	4.6	0.003*
Residence without fence	8	18	30.7		

* Significant association

Our study also showed that 51.4% of dogs kept in fenced residences were vaccinated compared to only 26.9% of those kept in un-fenced residences. In DRC, the majority of residences of middle or high economic class households are usually fenced to prevent their dogs from roaming. It can be deduced that since dogs kept in fenced residences are prevented from roaming, they can easily be restrained for vaccination compared to dogs kept in un-fenced residences. The latter are difficult to catch for vaccination purposes. However, the difference in the vaccination rate observed between fenced and unfenced residences may equally be due to the socio-economic level of the dog-owners rather than the physical barriers that prevent dogs from roaming.

The knowledge factors significantly associated with dog rabies vaccination were: Knowledge of rabies (OR = 8.0, p<0.05) and knowledge of location of veterinary office or service (OR = 3.4, p<0.05). These results demonstrate that rabies awareness or knowledge can increase rabies vaccination coverage. It was observed that 39% of dog owners did not vaccinate their dogs due to ignorance of location of veterinary offices or clinics and 40% due to negligence. Reported negligence seems to be high but this could have been confounded by their ignorance of rabies. In fact, only 14% of respondents reported a good knowledge of rabies “Table 2”.

**Table 2:** Knowledge factors associated to dog rabies vaccination.

Factors	Dog rabies vaccination				
	Yes	No	Proportion(%)	OR	P value
**Knowledge of rabies**	21	2	91.2	8	0.0004*
Yes	81	62	56.6		
No					
**Knowledge of location of veterinary offices / clinics**					
Yes	48	13	78.7	3.4	0.0002*
No	54	51	51.4		

* Significant association

Among the dogs’ characteristics, gender (OR = 1.6, p<0.05) and breed (OR = 2.1, p<0.05) were significantly associated with rabies vaccination “Table 3”. In the majority of African countries (up to 98%), dogs are used for socio-economic purposes: livestock protection against predators, protection against intruders, hunting and as a source of protein [14]. In Lemba, dogs are mainly used for guarding (95%) and consequently are usually aggressive breeds. Male dogs are more aggressive and much preferred than female dogs. In Chad, 80% of dog bites in humans originated from male dogs [19]. Bearing in mind the risk of biting people, owners of male dogs are prompt to have them vaccinated. Another reason for more male dogs being vaccinated than females is that they frequently fight for females during the mating season [20]. These fights can promote the transmission of rabies within the dog population and lead to culling which could be costly for owners of male hybrid dogs. This study showed that 59% of hybrid dogs were vaccinated compared to 42% of local dog breeds (mongrels) that were vaccinated. In Kinshasa local dog breeds cost less to buy than exotic or hybrid dog breeds (about USD 400 / puppy). Consequently, hybrid dog owners value their dogs more and are willing to pay for the cost of vaccination which is far lower than the price of the animal.

**Table 3:** Dog characteristics associated to rabies vaccination.

Factors	Dog rabies vaccination				
	Yes	No	Proportion(%)	OR	P Value
**Age groups**					
>1 year	65	58	52.8	1.4	0.13
≤1 year	43	52	42.5		
**Sex**					
Male	75	63	54.3	1.6	0.04
Female	34	46	42.5		
**Dog breed**					
Exotic and hybrid	52	33	59	2.1	0.003*
Local	56	77	42.1		
**Dog source**					
Bought	80	72	52.6	1.5	0.08
Other (owned dog progeny,gift	28	38	42.4		

* significant association

## Conclusion and policy perspectives

In this study, the DRC rabies vaccination coverage following the "owner-charged" scheme was higher (49.5%) than in other African countries. The coverage found in the study area can easily reach the 70% vaccination threshold recommended by WHO if a combination of mass dog vaccination campaign (“free of charge” scheme) as applied during rabies outbreaks and reduction of rabies vaccination cost can be applied towards increasing willingness-to-pay by low income dog owners. Increased knowledge on rabies control by dog owners and the accessibility of veterinary clinics are among critical factors decision-makers need to address in order to improve the level of vaccination coverage. This would, in turn, reduce human rabies incidence. Since this study was conducted in a limited area in Kinshasa, we recommend that similar studies be undertaken on a larger scale either in the Kinshasa itself where the disease remains endemic or in other provinces of the country for a better understanding of the parameters underlying the persistence of rabies in the DRC.

## Supporting information

S1 File Database generate from EPIINFO_Kazadi. (XLSX)

S2 File Survey questionnaire–French. (DOCX)

S3 File Survey questionnaire_determinants of dog rabies vaccination. (DOCX)
