# Supplementary material for: Determinants of dog owner-charged rabies vaccination in Kinshasa, Democratic Republic of Congo
Source: PLoS One. 2017 Oct 23;12(10):e0186677. doi: 10.1371/journal.pone.0186677 (PMC5653331; doi:10.1371/journal.pone.0186677)
Supplement: S2 File — (DOCX) [file pone.0186677.s002.docx]

#### N° Ménage…../…..

**Questionnaire enquête sur les déterminants de la vaccination antirabique des chiens**

Nom enquêteur ……………………… (Réservé à l’investigateur) Date de l’enquête…. / ……. /…….

Bonjour / Bonsoir. Mon nom est …………………………….. Je suis *(Titre/ Poste et lieu de travail)……………….* Nous menons une étude visant à étudier les déterminants de la vaccination antirabique des chiens dans votre zone de santé. Nous aimerions vous poser quelques questions relatives à cela. Toute information de notre entretien sera traitée de façon strictement confidentielle et les résultats de cette étude seront utilisés pour aider à prévenir la rage. Vous êtes libre de quitter l’étude. Etes-vous d’accord de participer à l’étude ?

**Section 1 : Informations sociodémographiques du répondant**

1. Âge ………….ans 2. Sexe : Masculin  Féminin

3. Quartier :
6. Niveau d'instruction : Sans niveau Primaire Secondaire Supérieur Pas de réponse

7. Profession : ……………………………………………………………………………………………….

8. Nombre pièces de la maison : 1 à 2 pièces Plus de 2 pièces

9. Votre maison est-elle électrifiée ? Oui Non Je ne sais pas

10. Disposez-vous d’un robinet dans la parcelle ? Oui Non Je ne sais pas

11. Moyen de locomotion chef de ménage ? Voiture privée Transport en commun Autres

12. Type de parcelle:

Clôturée permettant le confinement du chien Non clôturée

Clôturée ne permettant pas le confinement du chien

13. Nombre de personnes vivant dans le ménage :…………………………………………………………

**Section 2 : connaissances sur la rage et services vétérinaires**

14. Avez-vous déjà entendu parler de rage ? Oui Non Je ne sais pas

**Si la réponse est Non, passer à la section 3.**

15. Si oui, par quel moyen ?

A la télévision A la radio A la presse Par le voisin A l’école

Autre (à préciser) :………………………………………………………………………………………………………………............

16. Pouvez-vous citer les signes de rage chez l’homme ?

Etat d’excitation Paralysie Peur de l’eau Fièvre Toux Diarrhée

Démangeaisons ou fourmillement à l’endroit de morsure

17. Pouvez-vous citer les signes de rage chez le chien ?

Agressivité Paralysie Peur de l’eau Bave Agitation Décès

Perte d’appétit Autre (à préciser) : …………………………………………………………………………………….

18. Comment peut-on attraper la rage ?

Par morsure d’un animal enragé Par la sorcellerie

En mangeant la viande de chien En mangeant la viande d’un animal enragé Par griffure d’un animal enragé Par la salive d’un animal enragé lorsqu’un animal enragé vous lèche la plaie Autre (à préciser) :………………………………...

19. Selon vous, quels sont les animaux qui peuvent transmettre la rage ?

Le chien Le chat Le singe La chauve-souris Les souris

Autres (à préciser) :………………………………………………..............................................................

20. Connaissez-vous le moyen de prévenir la rage ? Oui Non Je ne sais pas

21. Si oui, lesquels ?...............................................................................................................................

22. Connaissez-vous l’existence des services vétérinaires ? Oui Non Je ne sais pas

23. Si oui, lesquels?.........................................................................................................................

**Section 3 : Informations sur le chien**

24. Avez-vous des chiens dans votre ménage ? Oui Non Je ne sais pas

25. Si non, pourquoi ?...........................................................................................................................

26. Si oui, combien ?............

**Si le ménage a plus d’un chien, mentionner le nombre au dessus de chaque catégorie (sexe, âge, race, source).**

27. Propriétaire : Chef ménage Autres adultes mâles Autres adultes femelles

Enfant Autre

28. Sexe: Male Femelle

29. Age: < 1 an 1 - 2 ans 3ans 4 ans 5 ans et plus

30. Race: Locale Croisée Exotique

31. Source chien: Don Achat Ramassé Voisin Propre niche Autre :……………………….

32. Mode élevage : Errant Confiné Mixte

33. Utilité : Compagnie Gardien Autres :……………………………………………………..

34. Est-ce qu’au moins un chien est vacciné contre la rage? Oui Non Je ne sais pas

35. Par qui ? Vétérinaire privé Vétérinaire du secteur public Autre (à préciser) ………………….

36. Où a-t-il été vacciné?...........................................................................................................................

| 37. A quand revient la dernière vaccination antirabique de votre chien?  Moins d’une année passée Plus d’une année passée Jamais |
| --- |
| 38. Avez-vous un certificat de vaccination ? Oui Non Je ne sais pas  39. Ce carnet est-il valide ? Oui Non |

40. Si au moins un de vos chiens n’est pas vacciné contre la rage, quelles sont les raisons?

Coût vaccination élevé Négligence Ignorance des services vétérinaires Etatiques et privés Autre (à préciser) :……………………..
