# Supplementary material for: Determinants of dog owner-charged rabies vaccination in Kinshasa, Democratic Republic of Congo
Source: PLoS One. 2017 Oct 23;12(10):e0186677. doi: 10.1371/journal.pone.0186677 (PMC5653331; doi:10.1371/journal.pone.0186677)
Supplement: S3 File — (DOCX) [file pone.0186677.s003.docx]

#### N° household…../…..

**Survey questionnaire on determinants of dog rabies vaccination**

Name of the investigator ……………………… Date of investigation …. / ……. /…….

Hello! My name is …………………………….. I am *(*position*)……………….* The main goal of our study is to identify the determinants of dog rabies vaccination in your area. I want to ask you some questions about it. Any information collected from this interview will be protected and the results will be used for rabies prevention and control. Please, keep in mind that your are free to opt out of the survey. Are you willing to participate to this survey?

**Section 1 : Socio-demographics characteristics of household**

1. Old ………….year 2. Gender: Male   Female

3. Quartier:
6. Formal education level: None Primary Secondary Tertiary No response

7. Employment status: ……………………………………………………………………………………………….

8. Number of bedroom: 1- 2 > 2

9. Availability of electricity? Yes No I don’t know

10. Availability of piped drinking water? Yes No I don’t know

11. Head household means of transportation? Private vehicule Public vehicule Others

12. Type of residence: Residence with fence Residence with fence that partially or not restricted dog movement Residence without fence

13. Household size:…………………………………………………………

**Section 2: Knowledge on rabies disease and veterinary offices**

14. Did you hear about rabies? Yes No I don’t know

**If no, skept to the section 3.**

15. If yes, how?

Television Radio News papers Community School Other

16. Clinic manifestations of human rabies?

Excitation Paralysisis Hydrophobia Fever Cough Diarrhea

Pruritis from the lesion

17. Clinic manifestations of dog rabies?

Agressivitity Paralysisis Hydrophobia Dog drool Excitement Death

Anorexia Other: …………………………………………………………………………………….………………………………

18. How rabies is transmitted?

Bite from rabid dog Witchcraft Consumption of dog meat

Consumption of rabies dog meat scratches from rabid dog

Saliva from rabid dog Lick from rabid dog Other:………………………………...

19. Animal whose can transmit rabies?

Dog Cat Non human primate Bat Mouse Others :………………………………………

20. Do you know the method used to control rabies? Yes No I don’t know

21. If yes, wich methods: ........................................................................................................................

22. Do you know the veterinary offices /clinics location? Yes No I don’t know

23.If yes, which?.....................................................................................................................................

**Section 3 : Informations on rabies**

24. Do you have dog in your household? Yes No I don’t know

25. If not, why ?...........................................................................................................................

26. If yes, how many? .............................................................................................................

27. Dog owner: Household head Other male major Other female major Child

28. Sex : Male Female

29. Old : < 1year 1 – 2 years 3 years 4 years 5 years and more

30. Breed: local C crossbred Exotic

31. Source of dog: Gift Bought Progeny of owned dog other

32. Dog confinement t: Free to roam Restricted

33. Dog usage: Pet Guard Other:……………………………………………………..

34. Did your dog vaccinated against rabies? Yes No I don’t know

35. Vaccinator? Veterinary Non veterinary

36. Where did he been vaccinated?........................................................................................................

| 37. Last vaccination period? One year earlier More than one year |
| --- |
| 38. Do you have the vaccination certificat ? Yes No I don’t know |

39. Reasons of non vaccination?

Too young for vaccination High cost of rabies vaccination Negligence

Ignorance of location of veterinary offices/clinics Other:…………………………………………………….
